# *Nannizzia polymorpha* as Rare Cause of Skin Dermatophytosis

**DOI:** 10.3201/eid2907.230477

**Published:** 2023-07

**Authors:** Pei-Lun Sun, Ching-Chi Chi, I-Hsin Shih, Yun-Chen Fan

**Affiliations:** Chang Gung Memorial Hospital Linkou Main Branch, Taoyuan, Taiwan (P.-L. Sun, C.-C. Chi, I.-Hsin Shih, Y.-C. Fan);; Chang Gung University, Taoyuan (P.-L. Sun, I-Hsin Shih, C.-C. Chi)

**Keywords:** *Nannizzia polymorpha*, fungi, fungal infections, dermatophyte, skin dermatophytosis, tinea capitis, tinea manuum, Taiwan

## Abstract

*Nannizzia polymorpha* is a dermatophyte that rarely infects humans. We describe 2 case-patients from Asia who had an inflammatory type of tinea capitis and tinea manuum caused by infection with this fungus. The diagnosis was confirmed on the basis of the morphologic and molecular characteristics of the microorganism.

Tinea is the most common fungal human skin infection caused by dermatophytes. It is caused by a group of fungi that can digest keratin in the stratum corneum of the skin and affects both humans and animals; animal-to-human transmission is possible. According to current molecular taxonomy, dermatophytes consist of 59 species in 9 genera (*1*). In addition to common pathogens, such as *Trichophyton rubrum* in humans and *Microsporum canis* in companion animals, several cases of sporadic infections caused by lesser-known dermatophyte species have been reported (*2**–**6*). We describe 2 cases of tinea caused by *Nannizzia polymorpha*, which were confirmed by using morphologic characteristics and molecular methods.

## The Study

This research has been approved by the Institutional Review Board of Chang Gung Medical Foundation (approval nos. 202001561B0 and 202300067B0). The patient consent was waived by the institutional review board.

Case-patient 1 was a 10-year-old boy who had abrupt onset of a scalp lesion that was 0.5 cm in diameter initially and evolved rapidly over the following 2 days. Physical examination showed a round erythematous plaque studded with multiple pustules and hair loss on his scalp measuring ≈3 cm in diameter. He had no history of animal or soil contact and had not visited a barbershop before the onset.

On the basis of a clinical diagnosis of tinea capitis, pus was collected for fungal and bacterial cultures, and treatment with oral griseofulvin (500 mg/d) was started. Nine days after treatment started, rashes appeared on the patient’s face, ears, trunk, arms, and legs. The treatment was changed to oral terbinafine (250 mg/d) because of a suspected allergic drug reaction. However, the rashes persisted, so his parents stopped treatment after 2 doses of terbinafine. The rashes disappeared thereafter, and the scalp lesion also resolved gradually during the following 2 weeks.

**Figure 1:**
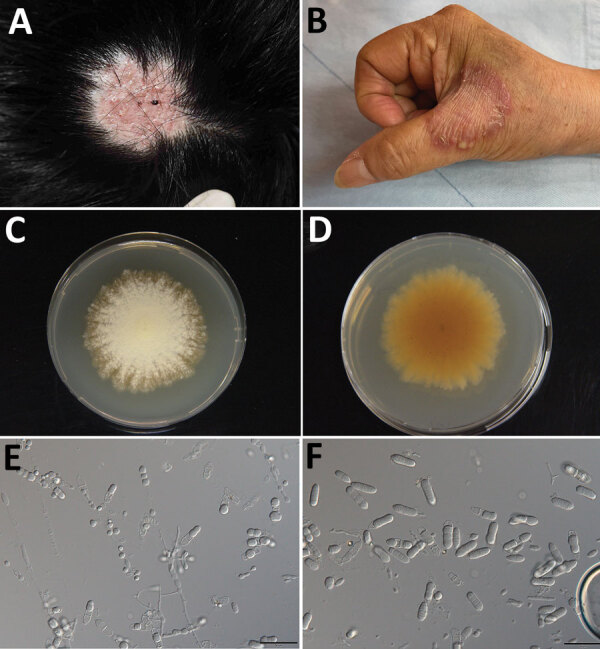
Clinical manifestations in 2 patients with rare cases of skin dermatophytosis caused by *Nannizzia polymorpha*. A) Case-patient 1, with tinea capitis; B) case-patient 2, with tinea manuum. C, D) The colony of isolate CGMHD 3492 isolated from case-patient 1 on potato dextrose agar obverse (C) and reverse (D). E, F) Microscopic features of microconidia (E) and macroconidia (F) (scale bars = 50 μm).

At a follow-up visit, the residual lesion was a round erythematous hairless patch (Figure 1, panel A). Two residual broken hairs were noted by dermoscopy but were negative for fungal spores on microscopy. Fungal culture was performed on a sample collected by using a sterile shampoo brush, but the result was negative.

Case-patient 2 was a 68-year-old woman who had a skin lesion on her right hand that had persisted for >1 month. The lesion had been treated with oral griseofulvin, topical tolnaftate, and econazole at a local clinic but had progressed. The patient had no history of soil or animal contact. The erythematous patch on her right hand had raised borders studded with scales and tiny, itching, nontender pustules along the border (Figure 1, panel B). The diagnosis of tinea manuum was confirmed by the presence of hyaline hyphae and arthroconidia on microscopy. The lesion responded to concomitant treatment with oral terbinafine (250 mg/d) for 2 weeks and topical 1% ciclopirox cream. Mycologic cure was achieved in 4 weeks.

The fungi isolated from both patients were morphologically similar. On potato dextrose agar, the colonies were light yellow with a lanose surface and radiate border (Figure 1, panel C) and an orange-yellow reverse surface (Figure 1, panel D). Microscopy showed abundant polymorphic microconidia and macroconidia (Figure 1, panels E, F). The macroconidia were borne on short conidiophores, moderately thick-walled, cylindrical, clavate, or ellipsoidal, with a rounded tip and tapered base, 1–4-septate, and 9–16 × 15–37.1 μm. The microconidia were sessile or short-stalked, 1-celled or occasionally 2-celled, clavate or tear-shaped with a truncate base, and 2.9–7 × 5–11.5 μm. The abundant intercalary conidia were barrel-shaped, spathulate, or cylindrical. The intercalary chlamydospores were spherical, borne singly, in pairs or occasionally in chains, and 4.9–8.7 μm in diameter.

**Figure 2:**
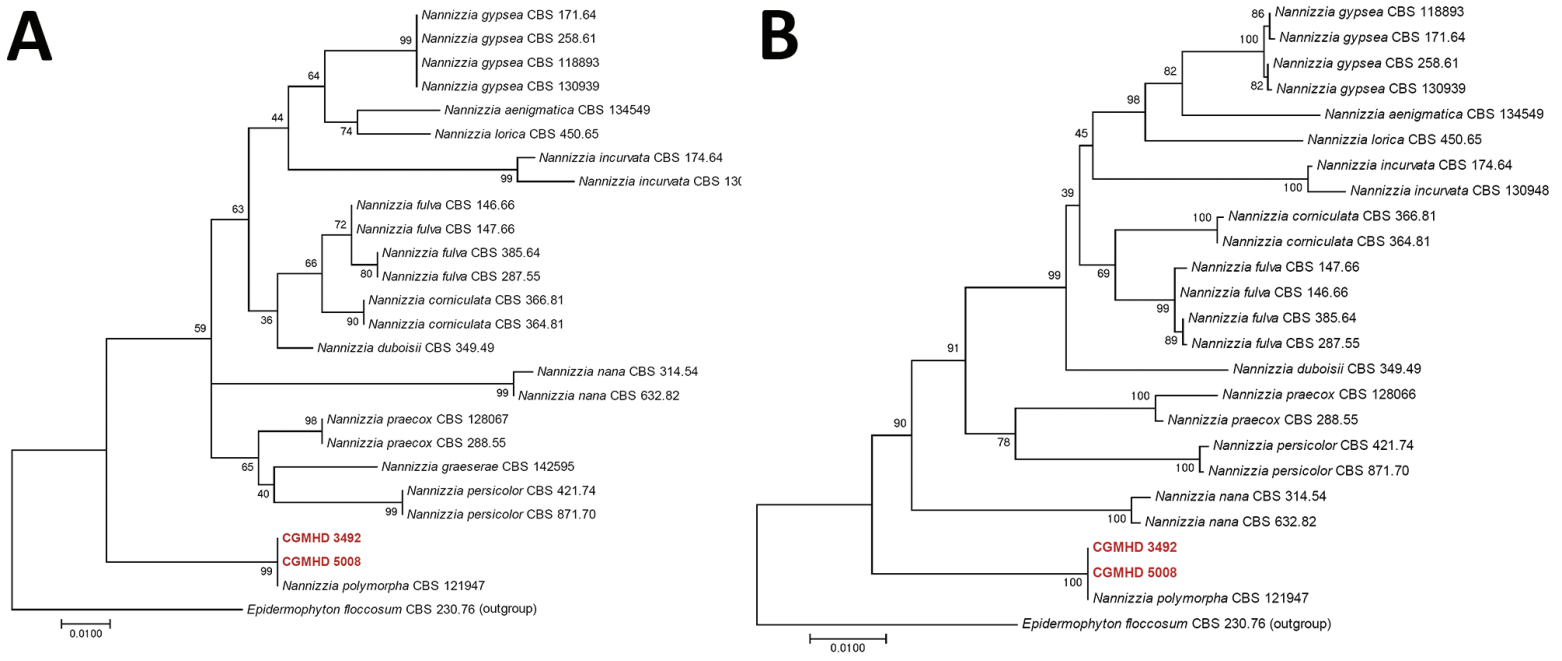
Phylogenetic trees of *Nannizzia polymorpha* in 2 patients with rare cases of skin dermatophytosis caused by this fungus. Trees were constructed by using MEGA 7.0 software (https://www.megasoftware.net) and the maximum-likelihood method on the basis of the internal transcribed spacer region (A) and combined datasets of internal transcribed spacer region, large subunit of ribosomal DNA, partial β-tubulin gene, translation elongation factor 3, and 60S ribosomal protein L10 (B). Red indicate strains isolated in this study. Tamura-Nei was used as a substitution model in both analyses. Numbers at nodes are bootstrap values. Scale bars indicate nucleotide substitutions per site.

The sequence of the internal transcribed spacer (ITS) of ribosomal DNA of the isolate had a 99.37% similarity with that of *N. polymorpha* strain CBS 121947. Phylogenetic analysis using ITS alone and multilocus analysis using ITS, large subunit of ribosomal DNA, partial β-tubulin gene, translation elongation factor 3, and 60S ribosomal protein L10 confirmed that both isolates were *N. polymorpha* (Figure 2). 

The sequences were deposited into GenBank (accession nos. OQ422181 and OQ427395 for ITS, OQ422485 and OQ427396 for large subunit of ribosomal DNA, OQ538298 and OQ538302 for 60S ribosomal protein L10, OQ538303 and OQ538301 for translation elongation factor 3, and OQ538299 and OQ538300 for partial β-tubulin gene).

We determined the MICs for 8 antifungal agents of the 2 isolates (Table) by using Clinical and Laboratory Standards Institute M38 Reference Method for Broth Dilution Antifungal Susceptibility Testing of Filamentous Fungi protocol for dermatophytes (*7*). 

**Table:** MICs of antifungal agents for the 2 *Nannizzia polymorpha* isolates implicated in rare cases of skin dermatophytosis

Antifungal agent	MIC, μg/mL
Fluconazole	2–4
Griseofulvin	0.125–0.25
Terbinafine	0.063–0.125
Itraconazole	0.063–0.25
Isavuconazole	0.016–0.031
Voriconazole	0.031–0.063
Efinaconazole	<0.008
Luliconazole	<0.008

## Conclusions

*Nannizzia* is a genus that had included the sexual stages of certain dermatophytes. In the latest classification of dermatophytes proposed in 2017, it is now the genus name for most geophilic and a few zoophilic fungi from the genus *Microsporum* (*8*). There are currently 13 species in the genus *Nannizzia*. Human infections by *Nannizzia* are opportunistic and result from contact with contaminated soil. Most cases are caused by *N. gypsea* species complex (*N. gypsea*, *N. incurvata*, *N. fulva*), and the clinical manifestations are tinea corporis, tinea capitis, and onychomycosis. Other members of the genus *Nannizzia* that have been reported to cause human infections include *N. aenigmatica* (*9*), *N. duboisii* (*9*), *N. lorica* (*2*), *N. nana* (*4*), *N. perplicata* (*10*), *N. persicolor* (*4*), *N. praecox* (*6*), and *N. polymorpha*.

*N. polymorpha* is a rare dermatophyte species. The type strain CBS 121947 was originally called *Microsporum amazonicum*. However, later morphologic and phylogenetic studies showed that it was new, and the species name *N. polymorpha* was proposed in 2019 (*8*). The strain was isolated from facial lesions of a patient in Kourou City, French Guiana (*11*). No additional cases or isolation records have been reported to our knowledge.

Case-patient 1 had an infection of the scalp accompanied by a prominent inflammatory reaction and hair loss. Although microscopic examination was not performed initially, the pathogenic role of the fungus in this patient was established by the massive growth of a single pure fungus and the good response to antifungal treatment. Case-patient 2 had tinea manuum and a prominent inflammatory reaction. Both patients responded to short-term antifungal treatment. Although there are currently no clinical breakpoints for antifungal agents against *N. polymorpha*, it is probably susceptible to all 8 antifungal drugs tested because of the low MIC values.

To our knowledge, there have been no reports of environmental isolation or animal infections caused by *N. polymorpha*. Its rarity might be attributable to the paucity of environmental surveys on dermatophytes or because it was only named in 2019. All 3 isolates reported were from patients with skin infections. The inflammatory characteristics of the lesion suggest that it is geophilic. However, further studies are needed to elucidate its ecologic niche.

Laboratory diagnosis of *N. polymorpha* is simple because of the abundant and variable shapes of macroconidia and microconidia and the typical cylindrical macroconidia with a blunt rounded apex. The colony was light yellow and lanose, which differentiated it morphologically from the cinnamon granular surface of species in the *N. gypse*a complex. *N. graeserae* has macroconidia similar to those of *N. polymorpha*; however, the ITS sequences showed only 523/589 (89%) similarity between our strain and *N. graeserae* CBS 142595. *Arthroderma flavescens* also has similar macroconidia, but they are longer than those of *N. polymorpha*, more monotonous, and uniform in shape. When an unidentifiable strain is encountered, DNA sequencing can provide an unequivocal diagnosis.
